# Effectiveness of acupuncture on anxiety disorder: a systematic review and meta-analysis of randomised controlled trials

**DOI:** 10.1186/s12991-021-00327-5

**Published:** 2021-01-30

**Authors:** Xiang-yun Yang, Ning-bo Yang, Fang-fang Huang, Shuai Ren, Zhan-jiang Li

**Affiliations:** 1 The National Clinical Research Center for Mental Disorders & Beijing Key Laboratory of Mental Disorders, Beijing Anding Hospital, Capital Medical University, No. 5 Ankang Hutong Deshengmen Wai, Xicheng District, Beijing, 100088 China; 2 Advanced Innovation Center for Human Brain Protection, Capital Medical University, Beijing, China; 3 First Affiliated Hospital of Henan University of Science and Technology, Luoyang, China; 4 School of Basic Medical Sciences, Henan University of Science and Technology, Luoyang, China; 5 Luoyang Fifth People’s Hospital, Luoyang, China

**Keywords:** Acupuncture, Anxiety disorder, Anxiety symptoms, Generalised anxiety disorder, Meta-analysis

## Abstract

**Background:**

A number of studies have shown the positive effects of acupuncture on state anxiety. However, the efficacy of acupuncture in treating anxiety disorder remains unclear. This review and meta-analysis aimed to explore whether acupuncture has a positive effect on anxiety disorder.

**Methods:**

Randomised controlled trials (RCTs) published in English and Chinese were found through various electronic databases, including PubMed, Scopus, the Cochrane Central Register of Controlled Trials, Embase, and the Chinese databases WanFang data, VIP Chinese Sci tech periodical database, and China National Knowledge Infrastructure. The primary outcome variable was extent of anxiety symptoms. The secondary outcomes included side effects and dropout rate. Effect sizes were pooled by random-effects modelling using Rev Man 5.3.

**Results:**

Twenty RCTs were included in this systematic review and meta-analysis. All included studies were designed for patients with generalised anxiety disorder (GAD), and 18 studies were published in Chinese. Egger’s test showed that the asymmetry of the funnel plot in all studies was not significant (*t* = − 0.34, *p* = 0.74). The meta-analysis of anxiety symptoms showed that acupuncture was more effective than the control condition, with a standard mean effect size of − 0.41 (95% CI − 0.50 to − 0.31; *p* < 0.001), and that acupuncture intervention showed good tolerance and safety in the treatment of anxiety disorder.

**Conclusion:**

Our findings suggest that acupuncture therapy aimed at reducing anxiety in patients with GAD has certain beneficial effects compared to controls. More RCTs with high quality should be conducted to fully understand the role of acupuncture in the treatment of various types of anxiety disorder. The protocol of this review was registered at the Prospero International Prospective Register of Systematic Reviews (Registration ID: PROSPERO 2020CRD42020148536).

## Background

Anxiety disorders constitute the largest group of mental disorders in most western societies and are a leading cause of disability [1]. New evidence shows that anxiety disorders are becoming a global problem, and that their prevalence is also increasing rapidly in developing countries [2, 3]. Anxiety disorder has become the most common mental disorder with a lifetime prevalence of 7.6% in China [4]. Individuals with anxiety disorder manifest both physical and mental symptoms, which may be chronic, and result in decreased ability, heavy economic burden, and poor quality of life [1].

Although the selective 5-HT reuptake inhibitors (SSRIs) and cognitive behavioural therapy (CBT) are the first-line treatment choice for anxiety disorder, the clinical methods used in the treatment of anxiety disorder are still insufficient, and the proportion of patients receiving appropriate treatment is low [5]. Moreover, even if patients with anxiety disorder receive sufficient first-line treatment, at least one-third of the patients still have poor treatment response [6]. More patients prefer complementary and alternative medicine (CAM) such as relaxation techniques, nutritional supplements, massage, and acupuncture, because they cannot tolerate the side effects of long-term medication [7]. Acupuncture is now commonly recognized as an effective CAM therapy, has been widely used to treat mental disorders, and has proven to have good tolerance and fewer side effects [8].

A number of studies have shown that acupuncture has a positive effect on situational anxiety related to operations, dental treatment, and examinations [9–12]. There are also several reviews and meta-analyses that have shown that acupuncture has positive effects on state anxiety [13, 14]. Diogo et al. [15] reviewed 13 studies published in English to explore the effectiveness of acupuncture for the treatment of patients with anxiety disorder, and found that acupuncture is an effective method with fewer side effects. However, they did not perform meta-analysis due to the heterogeneity of methods and poor experimental design in the included studies. Moreover, most of the studies in this review used the Hamilton Anxiety (HAMA) scale or the self-rating Anxiety Scale (SAS) to score the severity of anxiety disorders of patients rather than strict diagnostic criteria. It is worth noting that the anxiety level, duration of symptoms, and social functional impairment of patients who meet the diagnostic criteria of anxiety disorder are much higher than those of patients experiencing simple state anxiety. Therefore, it is difficult to determine whether acupuncture has a positive effect on anxiety disorders from that review.

The latest diagnostic criteria for anxiety disorders clearly define the classification of anxiety disorders, including panic disorder, phobias, social anxiety disorder, separation anxiety disorder, and generalised anxiety disorder (GAD) [1]. A number of clinical studies have proven that acupuncture is effective for anxiety disorders, especially for GAD in China [16]. However, few systematic studies have paid attention to the efficacy of acupuncture on anxiety disorders in a Chinese population. One study systematically reviewed the effect of acupuncture on GAD in a Chinese population and suggested that acupuncture was an effective and safe alternative therapy for GAD. But that review only included three studies whose results could not be combined because of their heterogeneity [17]. At present, there is not enough evidence to prove the efficacy and safety of acupuncture in patients with a definitive diagnosis of anxiety disorder.

To fill these gaps in the literature and to fully understand the effectiveness of acupuncture for treating primary anxiety disorder, we performed this meta-analysis by systematically reviewing relevant studies published in both English and Chinese. Our goal was to determine the efficacy of acupuncture for treating patients with definite anxiety disorder. We also considered the side effects and treatment compliance of acupuncture therapy.

## Methods

The protocol of this review was registered at the Prospero International Prospective Register of Systematic Reviews (Registration ID: PROSPERO 2020CRD42020148536, https://www.crd.york.ac.uk/prospero/display_record.php?ID=CRD42020148536). We performed this review and meta-analysis according to the Preferred Reporting Items for Systematic Reviews and Meta-analysis (PRISMA) guidelines and the recommendations of the Cochrane Collaboration.

### Literature search and selection

Two authors searched relevant literature from electronic databases including PubMed, Scopus, the Cochrane Central Register of Controlled Trials (CENTRAL), Embase, and the Chinese databases WanFang data, VIP Chinese Sci tech periodical database, and China National Knowledge Infrastructure (CNKI). The studies were restricted to peer-reviewed journal articles published between 2000 and August 2019. The date last searched was March 31, 2020. Searches were limited to those performed on human subjects and those written in English or Chinese. The keyword ‘acupuncture*’ was used in combination with ‘anxiety’ or ‘anxiety disorder’ or ‘general anxiety disorder’ or ‘panic disorder’ or ‘phobias’ or ‘social anxiety disorder’ or ‘separation anxiety disorder’ and ‘randomi*ed controlled trial’ or ‘controlled clinical trial’. The full PubMed search strategy is available in Additional file 1: Table S1.

We screened all relevant literature according to the inclusive and exclusive criteria. Study inclusion criteria were as follows: randomised controlled trials comparing acupuncture (including electroacupuncture) with a control condition (non-acupuncture, i.e. pharmacological treatment, sham acupuncture, other structured psychotherapies or standard care); use of standard diagnostic criteria to define anxiety disorder; both male and female adult subjects (age ≥ 18). Reduction of anxiety measured by accepted scales was the main outcome of interest. Studies that did not report specific anxiety scores were excluded.

After discarding duplicate studies, two authors (X.Y., N.Y.) independently screened the titles and abstracts of potentially eligible studies identified. Another two authors (F.H., S.R.) examined the full-text articles independently and determined whether they met the inclusion criteria. Any discrepancies were resolved through discussion with a third author.

### Data extraction

Two authors (X.Y., N.Y.) independently extracted data using data extraction forms, and two other authors checked the data. The data included study characteristics and participants (i.e., study population, sample size, age, gender, intervention form, delivery mode, acupoint of acupuncture therapy, condition of control group), and the outcomes measured from the selected studies. We selected anxious symptom, the core feature of anxiety disorder, as the main outcome. We also selected additional outcomes including dropout rate and side effects. For studies with follow-up, we extracted the results acquired at the end of treatment rather than after follow-up. The data of the Chinese studies were translated into English by two authors (X.Y., N.Y.) who are fluent in English and Mandarin. All data were re-checked for discrepancies and consensus was achieved by discussion.

### Assessment of risk of bias in included studies

Two authors independently assessed risk of bias for each study using the Cochrane Collaboration ‘Risk of bias’ tool, which consists of seven domains (sequence generation, allocation concealment, blinding of participants, personnel and outcome assessors, incomplete outcome data, selective outcome reporting, and other sources of bias). The risk of each domain was rated as high, low or unclear [18].

### Data analysis

Meta-analyses were conducted using RevMan analyses software (RevMan 5.3) [19]. This meta-analysis analysed per protocol analyses data of reduction of anxiety, because the measurement of anxiety was continuous data. We also analysed the intention-to-treat (ITT) data of subjects who had received intervention and were evaluated for symptoms of anxiety at least once after the intervention. A separate meta-analysis was performed for each outcome of interest. The included outcomes were reported in at least three studies. The standardized mean difference (SMD) was the effect measure for continuous variables, because the outcomes were measured using different scales. To calculate SMD, we used post-intervention means and their standard deviations. The random-effects meta-analysis was used.

Statistical heterogeneity of the RCTs was assessed using *I*^2^ statistics and its 95% CI with *R*. *I*^*2*^ > 50% or *p* < 0.05 indicated significant heterogeneity. A two-sided *p* ≤ 0.05 was considered significant. Potential publication bias was investigated using visual assessment of the funnel plot (plots of effect estimates against sample size) calculated by RevMan Analyses software. Publication bias may lead to asymmetrical funnel plots. We further quantified the asymmetry of funnel plots of studies using Egger’s test with *R*.

Subgroup analyses were conducted for anxiety symptoms to explore potential factors contributing to heterogeneity. The pooled mean difference was evaluated for each subgroup, and differences in mean differences between the subgroups (with a minimum of three studies) were examined. The subgroups were the type of anxiety assessment (clinician-assessment versus self-assessment); duration of acupuncture intervention (≥ 6 weeks versus < 6 weeks); and control condition (western medicine versus other Chinese traditional treatment).

## Results

### Literature search results

**Fig. 1:**
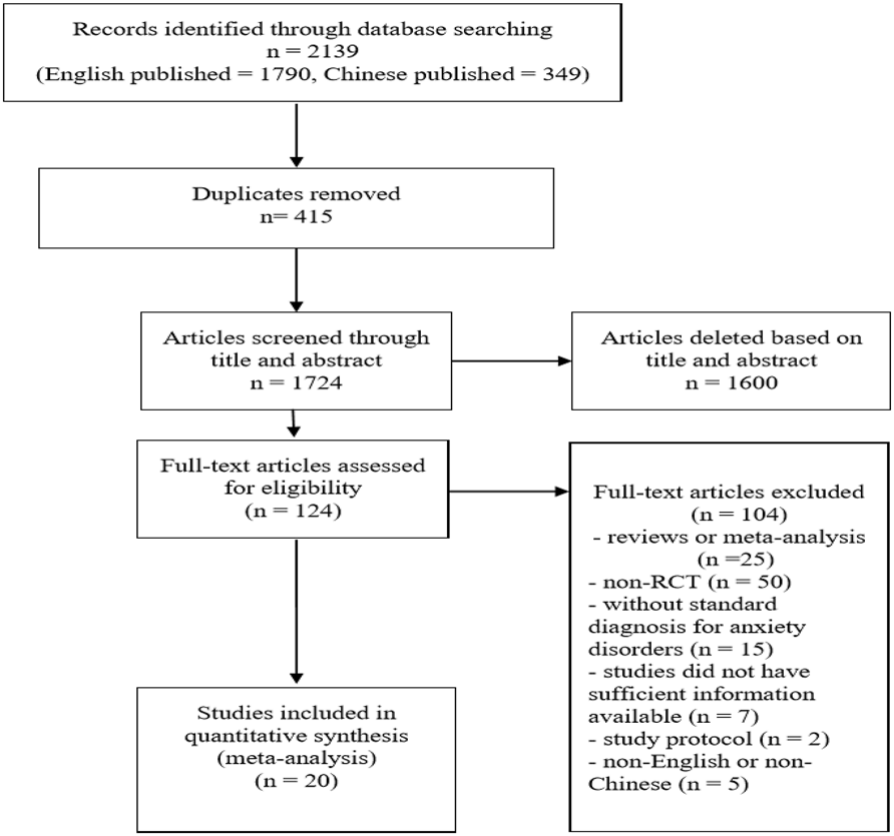
Flowchart for literature search and selection in this review and meta-analysis

The PRISMA flowchart (Fig. 1) shows the details of the literature search. The initial electronic search revealed 2139 studies, among which 415 duplicate articles were removed. Two assessors screened the title and abstracts of the remaining 1724 studies, and 1600 articles were then deleted, because they did not fulfil the inclusion criteria. Next, 124 full texts were examined carefully according to our inclusion and exclusion criteria. Finally, 20 RCTs were included in this systematic review and meta-analysis.

### Characteristics of included studies and participants

Full details of the characteristics of included studies are displayed in Table 1. Except for two studies published in English [24, 37], the rest were published in Chinese. All included studies were designed for patients with GAD [20–39]. The diagnosis of anxiety disorder in these studies was based on the criteria of diagnostic and statistical manual of mental disorders (DSM) or the third edition of Chinese classification of mental diseases (CCMD-3). All studies measured anxious symptoms before and after intervention, and two studies also measured the change of depressive symptoms. For the evaluation of anxiety symptoms, 15 studies used HAMA, a scale that needs to be evaluated by doctors, and 5 studies used self-assessment scales including SAS and 7-item Patient Health Questionnaire section for anxiety (PHQ-7). Two studies used electroacupuncture as an intervention, and others performed common acupuncture therapy. Sixteen studies chose western medicine including paroxetine, trazodone hydrochloride, and other anti-anxiety drugs as control. Three studies used other Chinese traditional treatment including self-acupoint massage, auricular point sticking therapy, and Chinese traditional medicine as controls. Only one study used sham acupuncture as control.

**Table 1:** Characteristics of included studies

Author (year, location)	Age (years) mean (SD)	Gender M/F	Number of subjects (I/C)	Diagnosis	Intervention methods & delivery (treated acupoints)	Type of control	Outcome measures used for meta-analysis	Report of side effect
Che, (2015, China)	43.2 (7.8)	32/48	80 (40/40)	GAD (CCMD-3)	Acupuncture Duration: 6 weeks Acupoints: Baihui, Shangxing, Shenting, Shuigou, Tanzhong, Juque, and Zhongwan	Medication: Paroxetine 20 mg/d, 6 weeks	Anxiety (HAMA)	TESS I: 6 cases C: 12 cases
Cheng et al. (2018, China)	I: 36.5 (8.6) C: 35.8 (7.9)	I: 19/14 C: 20/13	66 (33/33)	GAD (CCMD-3)	Acupuncture plus medication (Buspirone, same dose with control) Duration: 4 Weeks Acupoints: Baihui, Sishencong, Touwei, Shenmen, Neiguan, Shousanli, Sanyinjiao, and Yanglingquan	Medication: Buspirone 20 mg-30 mg/d 4 weeks	Anxiety (HAMA)	NR
Li et al. (2015, China)	I: 67(6.4) C: 69 (8.7)	I: 15/6 C: 17/7	45 21/24	GAD (CCMD-3)	Acupuncture Duration: 6 weeks Aucpoints: Laogong, Hegu, Neiguan, Shenmen, and Sanyinjiao	Medication: Lorazepam, 1.5 -3 mg/d Duration: 6 weeks	Anxiety (HAMA)	I < C
Liu et al. (2007, China)	38.48 (15.80)	30/56	86 29/28/29	GAD (CCMD-3)	Acupuncture Duration: 6 weeks Acupoints: Sishenzhen, Dingsehnzhen, Neiguan, Shenmen, and Sanyinjiao	C1*: medication: Paroxetine, 20 mg/d or fluoxetine 20 mg/d; or Alprazolam 0.4–1.6 mg/d C2: Acupuncture plus medication (dose same with C1)	Anxiety (HAMA)	TESS I < C2 < C1
Mak et al. 2019 (Hongkong, China)	I: 50.85 (11.57) C: 50.83 (14.15)	I: 20/20 C: 18/22	80 40/40	GAD (DSM-5)	Electroacupuncture Duration: 10 weeks Acupoint pairs: bilateral Neiguan and Shenmen, bilateral Zusanli and Shangjuxu, bilateral Sanyinjiao and LR3Taichong, and left Baihui and Yintang	Sham electroacupuncture; Utilize same acupoints with intervention group Duration: 10 weeks	Anxiety (7-item Patient Health Questionnaire section for anxiety); Depression (9-item Patient Health Questionnaire, PHQ-9)	NR
Wang et al. (2003, China)	I: 38.2 (9.4) C: 35.7 (8.8)	I: 7/13 C: 8/11	39 20/19	GAD (CCMD-3)	Electroacupuncture Duration: 6 weeks Acupoints: Yintang, Baihui, Xuanlu, and Fengchi	Medication: Trazodone Hydrochloride, 100–150 mg/d, Duration: 6 weeks	Anxiety (HAMA); Depression (SDS)	NR
Wang et al. (2005, China)	NR	NR	65 35/30	GAD (CCMD-3)	Acupuncture Duration: 30 days Acupoints: Yintang, Baihui, Neiguan, Shenmen, Tanzhong, and Sanyinjiao	Medication: Clomipram 0.5–2 mg, twice or three times a day, supplemented with Oryzanol 20 mg, three times a day, or Propranolol 10–20 mg, three times a day; Duration: 30 days	Anxiety (SAS)	NR
Wang (2007, China)	I, 18–63 C: 18–64	I: 9/12 C: 8/12	41 21/20	GAD (CCMD-3)	Acupuncture Duration: 30 days Acupoints: Bihui, Yintang, Shenmen, and Sanyinjiao	Medication: Deanxit, 2 tablets /d; Duration: 30 days	Anxiety (HAMA)	NR
Wang et al. (2010, China)	28–65	I: 11/19 C: 9/21	60 30/30	GAD (CCMD-3)	Acupuncture Duration: 3 weeks Acupoints: Xinyu, Ganyu, Danyu, Geyu, and Shenyu	Medication: Alprazolam, 0.4–0.8 mg/d Duration: 3 weeks	Anxiety (HAMA)	NR
Xiong, (2013, China)	16–55	32/39	71 36/35	GAD (CCMD-3)	Acupuncture Duration: 30 days Acupoints: Guanyuan, Zhongwan, Zhenwei, Tanzhong, Chengjiang, Baihui, Shenting, Fengfu, Dazhui, Zhiyang, and Mingmen	Medication: Deanxit, 2 tablets /d; and Oryzanol 30 mg/d Duration: 30 days	Anxiety (SAS)	NR
Xu et al. (2012, China)	I: 38(7.5) C: 39(8.4)	I: 12/20 C: 10/20	62 32/30	GAD (CCMD-3)	Acupuncture plus Chinese traditional medicine (dose was the same with the control group) Duration: 6 weeks Acupoints: Baihui, Sishencong, Yinquexue, Neiguan, Sanyinjiao, Hegu, and Taichong	Chinese traditional medicine: Shugan Qingre recipe: Chaihu, Huaimai, liquorice, rose, cicada clothing, and Scutellaria; One dose/d; Duration: 6 weeks	Anxiety (HAMA)	NR
Xu et al. (2016, China)	I, 25–60 C: 23–61	I: 14/16 C: 18/12	60 30/30	GAD (CCMD-3)	Acupuncture (Yu's scalp needle) Duration: 4 weeks Acupoint: the emotional areas, which are from the fontanelle to the shenting and its parallel lines of 1 inch and 2 inch to the left and right, respectively	Medication: Buspirone, 20–40 mg/d, 4 weeks	Anxiety (HAMA)	NR
Yan (2018, China)	I, 42.5 (4.2) C: 42.2 (4.0)	I: 13/17 C: 11/19	60 30/30	GAD (CCMD-3)	Acupuncture combined with self-acupoint massage Duration: 1 month Acupoints: Minghuangxue, Tianhuangxue, Qihuangxue, Baihui, and Yintang	Self-acupoint massage therapy: To press Fengchi point with both thumbs, temple point with left thumbs, Zhongwan point, Neiguan point, Qihai point, Tanzhong point with right thumbs, Baihui Point with right middle fingers. All the above acupoints were massaged by finger Duration: 1 month	Anxiety (HAMA)	NR
Zhang (2001, China)	16–60	160/130	296 157/139	GAD (CCMD-3)	Acupuncture Duration: 30 days Acupoints: Baihui, Neiguan, Renzhong, Sanyinjiao, and Beiyu	Medication: Doxepin, 150 mg/d; Duration: 30 days	Anxiety (SAS)	NR
Zhang et al. (2010, China)	I: 45.69 (5.54) C: 46.90 (5.89)	I:83/103 C:61/103	325 186/139	GAD (CCMD-3)	Acupuncture Duration: 4 weeks Acupoints: Three Huang Points of DONG’s extra-points (Minghuang, Tianhuang and Qihuang)	Medication: Buspirone, 15 mg/d; Duration: 4 weeks	Anxiety (HAMA)	I:1.6% C:33.8%
Zhao et al. (2014, China)	I: 42.31 (8.44) C: 41.5 (7.89)	I: 11/19 C: 13/17	60 30/30	GAD (CCMD-3)	Acupuncture Duration: 6 weeks Acupoints: Sishen, Congtou, Baihui, Shenting, Shuigou, Bneshen, Fengchi (bilateral), Neiguan (bilateral), and Shenmen (bilateral)	Medication: Fluoxetine, 20 mg/d; Duration: 6 weeks	Anxiety (HAMA)	TESS I: 2 cases C: 8 cases
Zhao et al. (2018, China)	I: 42.59 (8.17) C: 43.31 (7.96)	I:20/35 C:23/36	112 53/59	GAD (CCMD-3)	Acupuncture Duration: 6 weeks Acupoints: Yintang, Baihuim Fengchi, Neiguan, Sanyinjiao, and Taichong	Medication: Buspirone, 15 mg/d; Duration: 6 weeks	Anxiety (HAMA)	I: 3 cases C: 9 cases
Zheng et al. (2004, China)	I: 35 (5.2) C: 34(6.1)	I: 13/45 C:15/35	108 58/50	GAD (CCMD-3)	Acupuncture Duration: 8 weeks Acupoints: Xinyu, Jueyinyu, Baihui, Shenting, and Shenmen	Medication: Alprazolam, 0.4–0.8 mg/d; Duration: 8 weeks	Anxiety (HAMA)	I < C
Zheng et al. (2014, China)	I: 17–56 C: 22–53	I: 12/18 C: 9/21	60 30/30	GAD (CCMD-3)	Acupuncture Duration: 4 weeks Acupoints: Xinyu, Jueyinyu, Feiyu, Ganyu, Danyu,Weiyu, Piyu, Sanjiaoyu, Shenyu, and Baihui	Auricular point sticking therapy: Duration: 4 weeks	Anxiety (SAS)	NR
Zhou et al. (2013, China)	I: 38 (8) C: 38 (10)	I: 16/24 C18/22	80 40/40	GAD (CCMD-3)	Acupuncture Duration: 6 weeks Acupoints: Lieque, Hegu, Shenmen, Houxi, Waiguan, laogong, Yongquan, Zhibian, Yinlingquan, Zusanli, Taichong, and Yanglingquan	Medication: Clonazepam, 4–8 mg/d; Duration: 6 weeks	Anxiety (HAMA)	NR

*I* intervention, *C* control, *NR* Not Reported, *CCMD-3* Chinese standard for mental disorders and diagnosis (3rd Edition), *DSM* the diagnostic and statistical manual of mental disorders, *GAD* generalized anxiety disorder, *HAMA* Hamilton anxiety scale, *HAMD* Hamilton depression scale, *SAS* self-rating anxiety scale, *SDS* self-rating depression scale, *HSCL-25* self-rated Hopkins Symptom Checklist-25

^*^Selected as control group for those studies with two control groups

According to “Standards for Reporting Interventions in Clinical Trials of Acupuncture” (STRICTA), 12 of the included studies reported the types of needles used, including the diameter and length as well as the manufacturer and/or the material, and the others reported only the types of needles. All the studies reported the acupoints used, and the selection of acupoints was based on traditional acupuncture theory. Seventeen studies reported “de qi” sensations, where reportage of such was recommended.

### Risk of bias

**Fig. 2:**
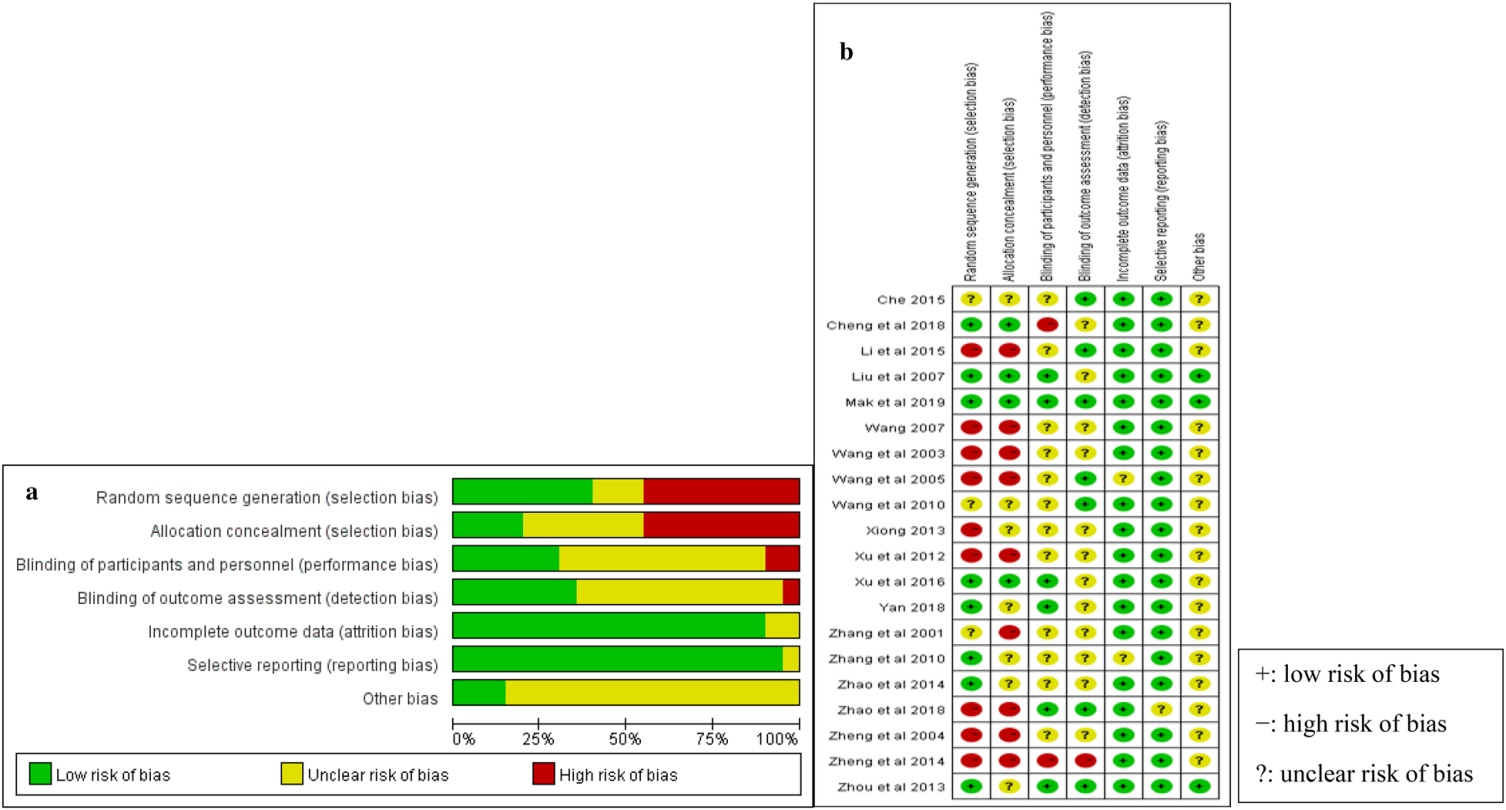
Risk of bias. **a** Each risk of bias item presented as percentages across all included studies. **b** Each risk of bias item in each study

The 20 included studies adopted parallel design, showing various degrees of bias susceptibility (Fig. 2). Only eight studies described a method of adequate randomization, the other four studies reported concealed allocation, although the word “randomisation” appeared in all the articles. Most studies did not describe how to prevent performance bias and detection bias. There was low risk on attrition bias and reporting bias in most studies.

### Effects of acupuncture therapy on anxiety symptoms

**Fig. 3:**
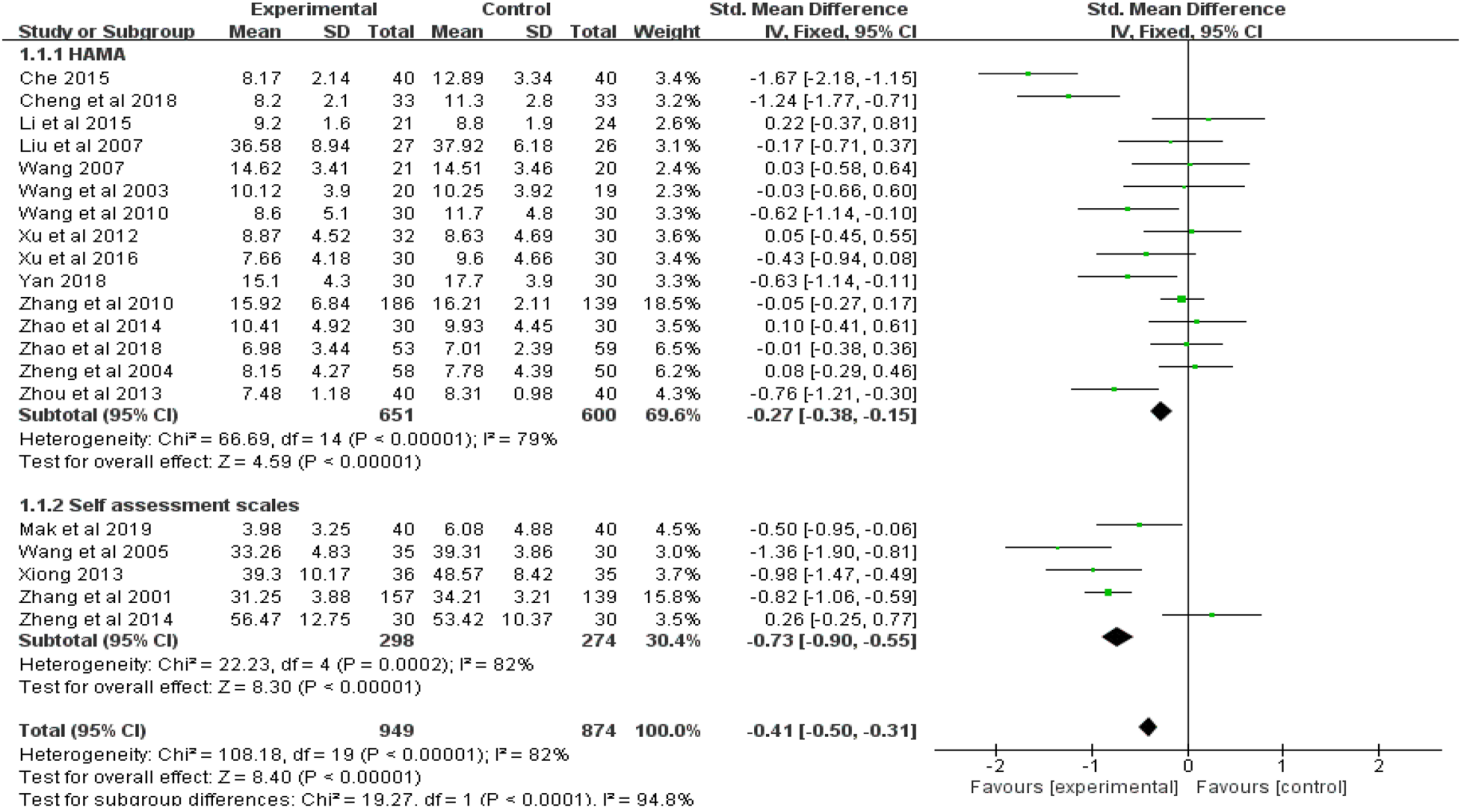
Forest plot of the effect of acupuncture on anxious symptoms in all studies and subgroups using different scales for anxiety assessment

**Fig. 4:**
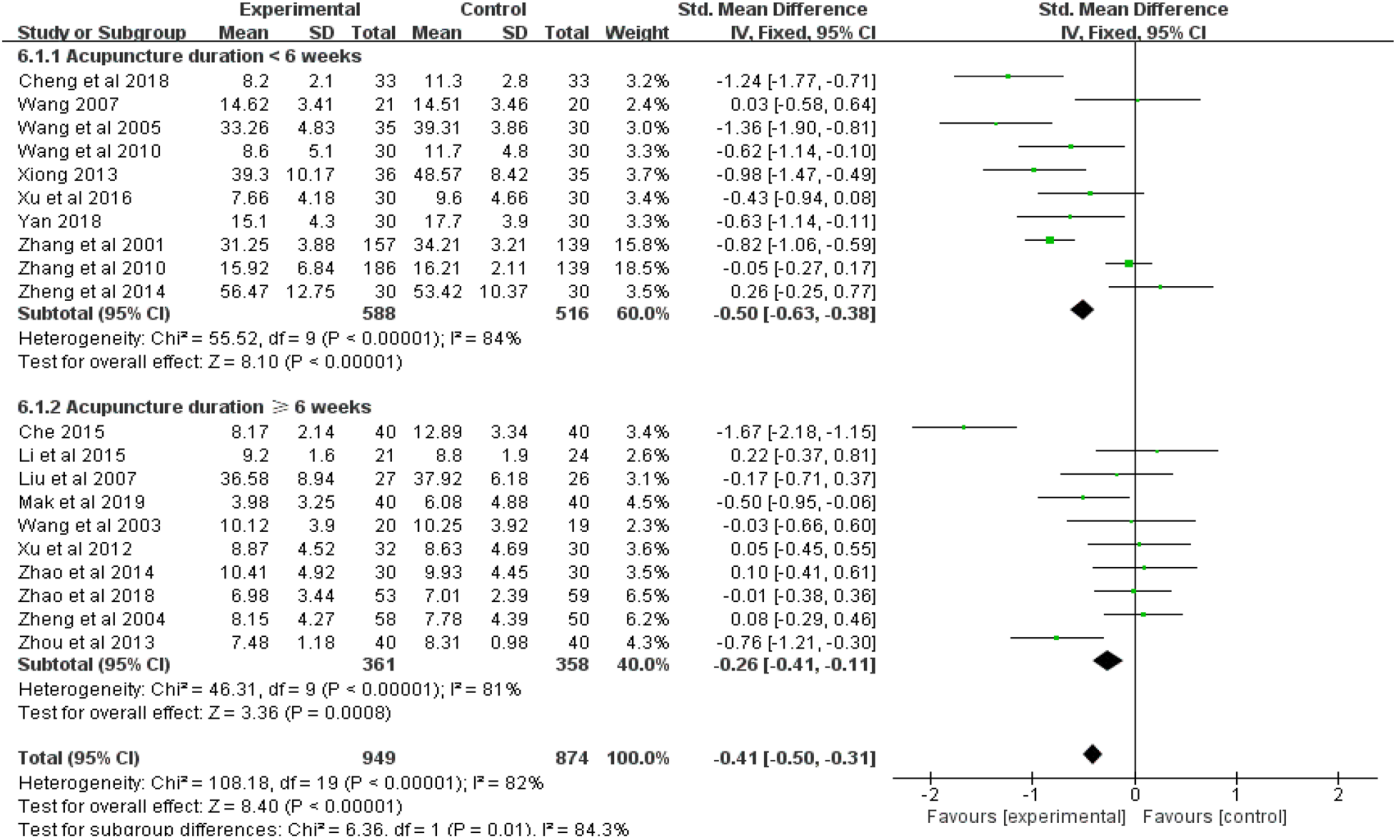
Forest plot of the effect of acupuncture on anxious symptoms in all studies and subgroups using different durations of acupuncture

**Fig. 5:**
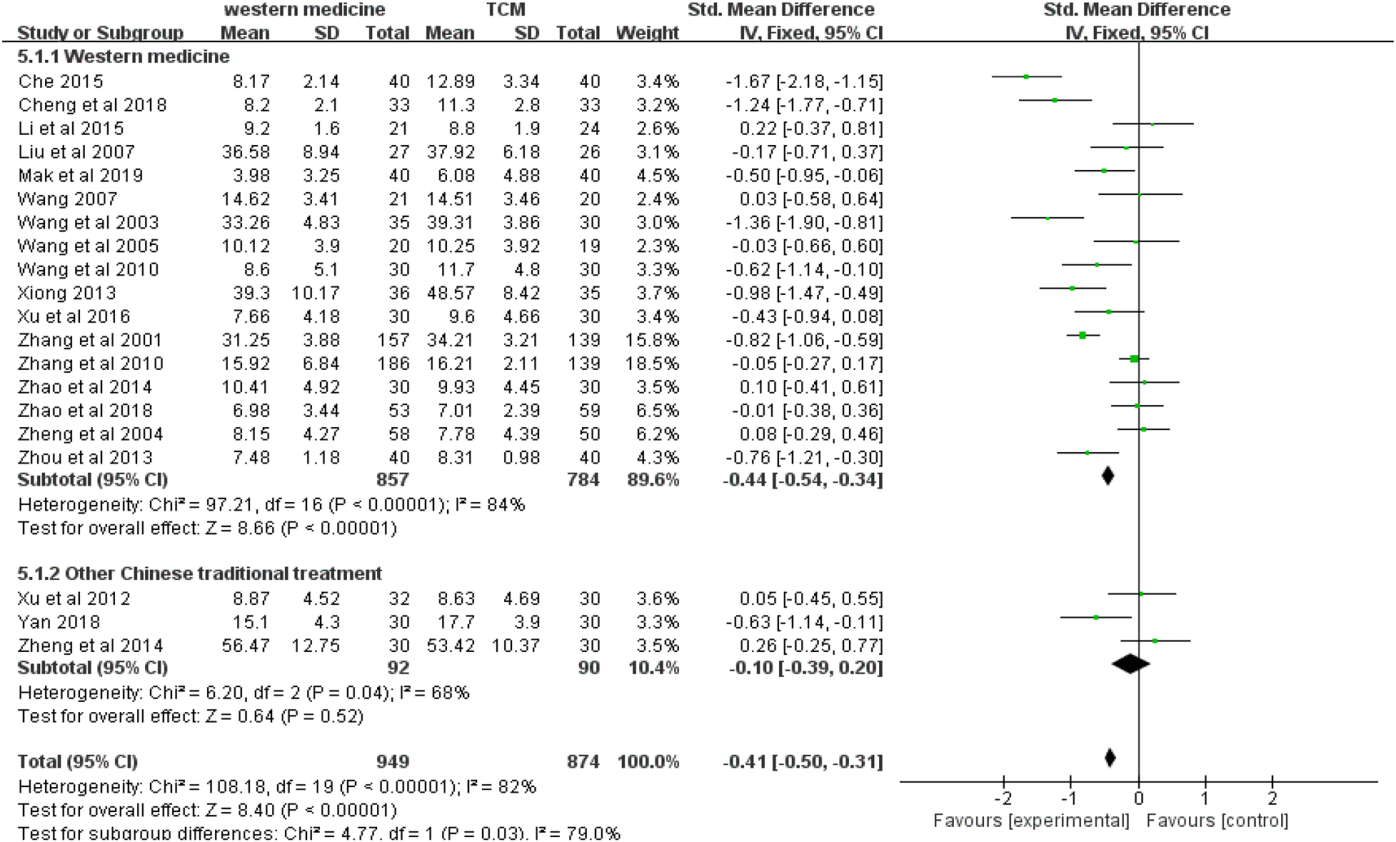
Forest plot of the effect of acupuncture on anxious symptoms in all studies and subgroups using different control conditions

Twenty studies (*n* = 1823) were included in the random-effects meta-analysis comparing the effect of acupuncture and control conditions on anxiety symptoms. Due to the lack of ITT data, we did the meta-analysis using per-protocol analyses data of all included studies. As shown in Figs. 3, 4 and 5, acupuncture was more effective than the control condition in reducing symptoms of anxiety, with a standard mean effect size of − 0.41 (95% CI − 0.50 to − 0.31; *p* < 0.001). The heterogeneity of these studies was statistically significant (*I*^2^ = 82%, 95% CI 0.74–0.83; *p* < 0.001).

Subsequent subgroup analyses were performed to explore potential factors contributing to heterogeneity. First, we conducted pre-planned subgroup meta-analyses for anxiety symptoms between two groups using either the clinician-assessment or the self-assessment scale to evaluate the reduction of anxiety. As shown in Fig. 3, acupuncture had better effect on reducing anxiety symptom than control condition in both groups, with a SMD of − 0.27 (95% CI − 0.38 to − 0.15; *p* < 0.001), and − 0.73 (95% CI − 0.90 to − 0.55; *p* < 0.001), respectively. The group using the self-assessment scale for anxious symptoms showed a better effect than the group using the clinician-assessment scale (*p* < 0.001). The heterogeneity of the studies in the group using the clinician-assessment scale was statistically significant (*I*^2^ = 79%, 95% CI 0.66–0. 87; *p* < 0.001). The heterogeneity of the studies in the group using the self-assessment scale was also statistically significant (*I*^2^ = 82%, 95% CI 0.59–0.92; *p* < 0.001).

Second, a subgroup meta-analysis for anxiety symptoms was performed between two groups with longer or shorter duration of acupuncture (≥ 6 weeks versus < 6 weeks). The results showed that acupuncture had a better effect on anxiety symptoms in both groups (see Fig. 4). However, the group treated with acupuncture for a shorter duration showed a better effect than the group treated for a longer duration (*p* = 0.01). The heterogeneity of two groups was also statistically significant (*I*^2^ = 81%, 95% CI 0.65–0.89, *p* < 0.001; *I*^2^ = 84%, 95% CI 0.72–0.91, *p* < 0.001).

Third, a subgroup meta-analysis for anxiety symptoms was performed between two groups using different control conditions (western medicine versus other Chinese traditional treatment). As shown in Fig. 5, acupuncture had a better effect on anxiety than western medicine with a SMD of − 0.44 (95% CI − 0.54 to − 0.34, *p* < 0.001). There was no significant difference between acupuncture and other Chinese traditional treatments (SMD: − 0.10, 95% CI − 0.39 to 0.20; *p* > 0.05). The heterogeneity of two groups was also statistically significant (*I*^2^ = 84%, 95% CI 0.75–0.89, *p* < 0.001; *I*^2^ = 68%, 95% CI 0.20–0.91, *p* < 0.001).

### Publication bias

**Fig. 6:**
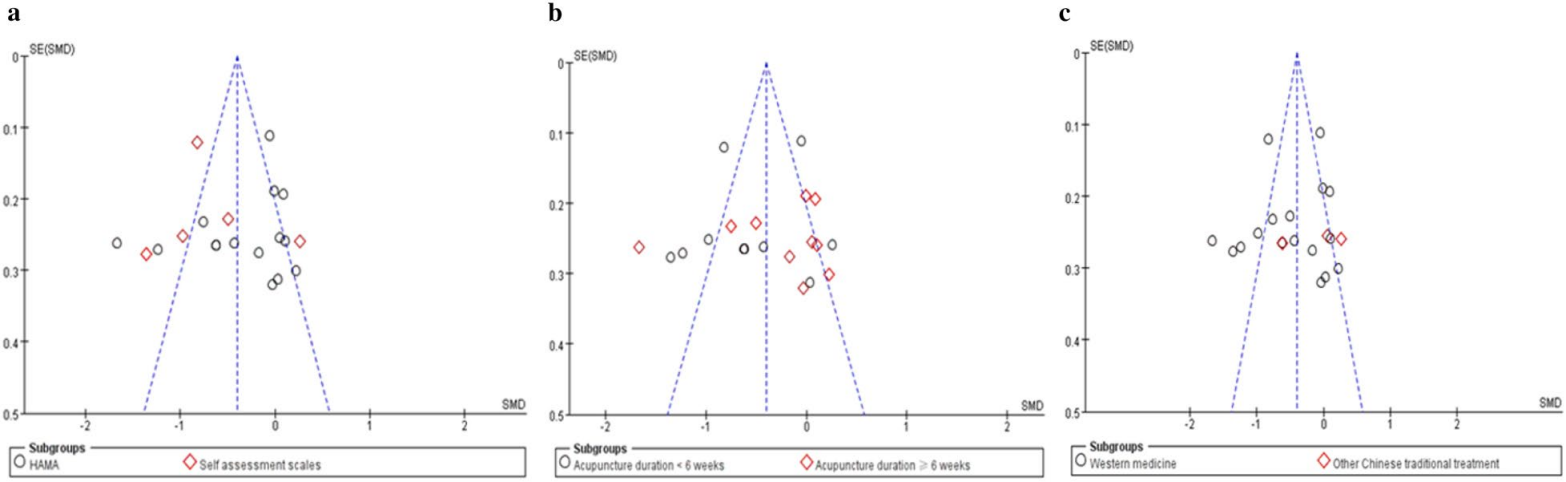
Funnel plots of all studies and subgroups. **a** The subgroups included the group using HAMA and the group using self-assessment for anxiety assessment. **b** The subgroups included the group with a longer duration of acupuncture treatment (≥ 6 weeks) and the group with a shorter duration of acupuncture treatment (< 6 weeks). **c** The subgroups included the group using western medicine as the control condition and the group using other traditional Chinese treatments as control conditions

Funnel plots of the primary outcome in all studies and subgroups are presented in Fig. 6. Visual assessment of the funnel plots suggests that publication bias is unlikely to affect the overall results of this meta-analysis. We further performed Egger’s test to quantify the publication bias. The results showed that the asymmetry of the funnel plot in all studies was not significant (*t* = − 0.34, *p* = 0.74). The asymmetry of the funnel plot in 15 studies using HAMA as anxiety assessment (*t* = − 1.19, *p* = 0.25), and 16 studies using western medicine as a control condition (*t* = − 0.57, *p* = 0.58) was also not significant. Due to the small number of studies in the group using the self-assessment scale for anxiety (*k* = 5) and in the group using other Chinese traditional treatment as control conditions, we were unable to test their specific publication bias. The publication bias of groups with longer acupuncture duration (≥ 6 weeks) and those with shorter duration (< 6 weeks) was not significant (*t* = − 0.72, *p* = 0.49; *t* = − 0.31, *p* = 0.77).

### Side effects and treatment compliance

Seven studies reported the occurrence of side effects during the intervention and showed that fewer side effects occurred in the acupuncture group compared to the control group. The most common adverse events during acupuncture were needle pain, hematoma, faintness during acupuncture treatment, and bleeding. Four studies reported that the dropout rate was less than 20% during acupuncture therapy, and it was lower or equal to the dropout rate for control groups. There was no measurement of anxiety in dropout subjects after intervention in those four studies. In summary, acupuncture intervention showed good tolerance and safety. Meta-analysis was not performed for the outcome variables concerning safety and compliance due to the lack of available data.

## Discussion

Anxiety is a core manifestation of anxiety disorder, which is characterized by persistent fear and despair, and is accompanied by the development of physical symptoms such as tachycardia, tension, pain, and inability to relax. Several reviews and meta-analyses have proven the positive effect of acupuncture on reducing state anxiety [8–11]. To our knowledge, there have been no other meta-analyses of RCTs conducted concerning the effectiveness of acupuncture on anxiety disorder. Our meta-analysis of 20 RCTs showed the effects of acupuncture on reducing anxious symptoms in patients with anxiety disorder. In addition, we conducted a subgroup meta-analysis to explore the possible factors affecting acupuncture efficacy and heterogeneity in all studies. Furthermore, our results showed that acupuncture is safe and has good compliance.

It is worth noting that all included studies examined the effectiveness of acupuncture on GAD. GAD is the most common anxiety disorder that is characterized by excessive, uncontrollable, unexplained, and often irrational worry. One previous systematic review that included only three RCTs suggested that acupuncture is an effective and safe alternative therapy for GAD [17]. In this study, we conducted meta-analysis of even more RCTs to further show the efficacy of acupuncture in the treatment of GAD.

Previous studies suggested that acupuncture may be used in emergency conditions involving emotional trauma, because the tools for acupuncture are conveniently portable, and acupuncture therapies are very cheap and safe [40, 41]. Anxiety disorder also comprises other psychiatric conditions such as panic disorder, phobias, social anxiety disorder, and separation anxiety disorder [1]. To our knowledge, few studies have explored the effect of acupuncture on other anxiety disorders except GAD [15, 16]. Thus, the current results cannot be over-interpreted to suggest a positive effect of acupuncture on all types of anxiety disorder. In the future, well-designed studies for more types of anxiety disorder are needed to expand the applications of acupuncture.

It should also be noted that all included studies were conducted in China. During the process of literature search, we found a number of studies conducted in Western countries that explore the effects of acupuncture on state anxiety [12, 13]. In contrast, most Chinese studies examined the efficacy of acupuncture for treating anxiety disorder. This phenomenon suggests that there is a different understanding and a different level of concern for state anxiety and anxiety disorder between Western countries and China. For a long time, Chinese people have neglected emotional problems, and only when the illness is serious enough to affect social functions do they pay attention to it [16]. Therefore, most of the patients who went to the hospital were diagnosed with anxiety disorder. Compared with medication, Chinese patients are more willing to accept acupuncture therapy because of its fewer adverse effects. Although different groups of people with varied cultural backgrounds have different acceptance of acupuncture, current findings provide evidence for the application of acupuncture in the treatment of anxiety disorder in more regions.

Subsequent subgroup analyses were performed to explore the different effects of acupuncture on anxiety in studies with different design. First, we performed a subgroup meta-analysis between two groups using clinician-assessment or self-assessment scales to evaluate the reduction of anxiety. The result showed that acupuncture has better effect on reducing anxious symptoms than control conditions in both the group using the HAMA scale and the group using the self-assessment scale. The effect in the group using self-assessment scales was greater than in the group using HAMA. This suggests that the effect of acupuncture on anxiety disorder might be overestimated using self-assessment scale to judge the reduction of anxiety symptoms, and that the participants’ subjective judgment might be not rigorous enough. To improve the accuracy and consistency of scoring the efficacy of acupuncture, clinician-assessment should be used to strengthen objective evaluations [42].

Second, a subgroup meta-analysis for anxiety symptoms was performed between two groups with longer or shorter duration of acupuncture (≥ 6 weeks versus < 6 weeks). We found that the group treated with acupuncture for a shorter duration showed a better effect compared to the group treated for a longer duration. This result, consistent with other studies, suggests that acupuncture works earlier and can quickly relieve anxiety within 6 weeks. However, anti-anxiety medicine and other interventions take effect slowly and showed efficacy after 6 weeks. Therefore, after 6 weeks, the effect of acupuncture was no different from that of other interventions. This finding suggests that that acupuncture can be used in the early stages of treatment for anxiety disorder, and can improve the first 6 weeks of the course of treatment.

Third, we performed a subgroup meta-analysis between two groups using different control conditions. The results showed that the group using western medicine as a control showed better effect of acupuncture on anxiety than the group using other Chinese traditional treatments as controls. The studies included in this meta-analysis only observed the short-term efficacy and did not follow-up the long-term efficacy. Thus, one reason for this result may be that western medicine takes effect slowly. Another reason for this result may be that Chinese people prefer to use traditional Chinese treatments, such as acupoint massage. It should be noted that there are only three studies using other Chinese traditional treatment, which also limits the reliability of this result. More research is needed to strengthen this finding.

High heterogeneity was observed in the pooled effects in this meta-analysis. Since significant heterogeneity is a common problem for continuous variables in meta-analysis, we chose the random effect model to combine the effects to partially eliminate the effect of heterogeneity. In subgroup analysis, we found that the effectiveness of acupuncture in reducing anxiety symptoms varied between groups with different study design. This suggests that the heterogeneity we observed may be due to the different measurement tools used for anxiety, different duration of acupuncture, and different set of control conditions. Therefore, considering the high heterogeneity, the results of this study need to be interpreted carefully.

Acupoints that are targeted for calming the mind, refreshing the brain and opening the orifices, soothing the liver, and strengthening the spleen were selected, including Baihui, Yintang, Sishencong, shenting, as were other head acupoints, including Neiguan, Xinshu, jueyinshu, Sanyinjiao, Zusanli, and Taichong, and other distal and back acupoints [16]. Generally, the acupoints selected in all studies were consistent in this meta-analysis. However, only two studies reported the manual of acupuncture therapy. Although the biological mechanism of acupuncture therapy remains unclear, some studies have provided evidence to explain the neuro-mechanism of acupuncture. In animal models, both behavioural and biochemical marker changes occur when acupuncture is used to reduce anxiety [6, 7]. In the future, more well-designed studies using a standard manual of acupuncture are needed to further confirm the efficacy of acupuncture in the treatment of anxiety disorder and to explore its biological mechanism.

A few of the studies reported side effects during interventions and suggested that acupuncture is relatively safer than control treatments. Consistent with other studies, the common adverse events during acupuncture therapy were needle pain, hematoma, faintness, and bleeding [43]. In all of the studies, the dropout rate of the acupuncture group was less than 20%, similar to that of the control groups. In summary, our findings show that acupuncture has good compliance and is safe for the treatment of anxiety disorder.

## Limitations

The present meta-analysis has several limitations. First, although we performed randomised effect analysis and subgroup analysis, the heterogeneity was significant in the pooled effects. Second, since all studies were performed in China, the representativeness of our current results may be insufficient due to the regional and ethnic restrictions. Third, all included studies explored the effectiveness of acupuncture on one kind of anxiety disorder—GAD. The effect of acupuncture on other types of anxiety disorder has not been reported. Moreover, most articles were published in Chinese; therefore, there may be a language bias. Potentially relevant papers in other important languages were not included in this review. Finally, we used text words instead of Medical Subject Headings as search strategy, and did not search manually, which may lead to incomplete search. Thus, current findings need to be considered carefully. In brief, more RCTs with high quality are needed to further prove the efficacy of acupuncture on anxiety disorder in different population in the future.

## Conclusion

In conclusion, this meta-analysis suggests that acupuncture therapy aimed at reducing anxiety in patients with GAD has certain beneficial effects compared to non-acupuncture therapy. More high-quality RCTs should be conducted to fully understand the role of acupuncture in the treatment of anxiety disorder.

## Supplementary Information

**Additional file 1.**

## Data Availability

All data generated or analysed in this study are included in this published article.

## Abbreviations

RCTs: Randomised controlled trials
CENTRAL: Cochrane Central Register of Controlled Trials
SMD: Standardized mean difference
CAM: Complementary and alternative medicine
HAMA: Hamilton anxiety scale
GAD: Generalised anxiety disorder
SAS: Self-rating anxiety scale
CCMD-3: Chinese standard for mental disorders and diagnosis (3rd Edition)
DSM: The diagnostic and statistical manual of mental disorders
HAMD: Hamilton depression scale
SDS: Self-rating depression scale
PHQ-7: 7-Item Patient Health Questionnaire section for anxiety
HSCL-25: Self-rated Hopkins Symptom Checklist-25
CBT: Cognitive behavioral therapy
CNKI: China National Knowledge Infrastructure
SSRIs: Selective 5-HT reuptake inhibitors
ITT: Intention-to-treat
STRICTA: Standards for Reporting Interventions in Clinical Trials of Acupuncture
