# Supplementary material for: Effectiveness of acupuncture on anxiety disorder: a systematic review and meta-analysis of randomised controlled trials
Source: Ann Gen Psychiatry. 2021 Jan 30;20:9. doi: 10.1186/s12991-021-00327-5 (PMC7847562; doi:10.1186/s12991-021-00327-5)
Supplement: Supplementary file 1 — Additional file 1. [file 12991_2021_327_MOESM1_ESM.docx]

**PRISMA-A条目清单（*修改条目；^●^新扩展的条目；其余为未改变的PRISMA条目）**

| **内 容** | **条 目** | | | | |
| --- | --- | --- | --- | --- | --- |
| ***标题*** |  | | |  | |
| 标题 | 1^*^明确报告研究是系统评价、Meta分析还是两者兼有；如果研究了具体的针刺类型，则应该在题目中说明，如手法针刺或电针。  Line 2 - 3 in page 1. | | | | |
| ***摘要*** |  | | |  | |
| 结构式摘要 | 2 提供结构式摘要：包括研究背景、目的、资料来源、纳入排除标准、研究对象和干预措施、研究评价和综合的方法、结果、局限性、结论和主要发现、系统评价的注册号。  Line 29 - 56 in page 2 - 3. | | | | |
| ***前言*** |  | | |  | |
| 理论基础 | 3^*^在背景中描述已知的针刺干预对目标疾病或症状的作用原理；若适用，需具体到拟研究的特定类型的针刺干预，并描述不同针刺类型之间的效果是否有差异。  Line 81-130 in page 4-5. | | | | |
| 研究目的 | 4 基于研究对象、干预措施、对照措施、结局指标和研究类型（PICOS）5个方面提出所需要解决的清晰明确的研究问题。  Line 131 - 136 in Page 5 - 6. | | | | |
| ***方法*** |  | | |  | |
| 方案和注册 | 5 如果已有研究方案，则说明方案内容并给出可获得该方案的途径（如网址），并且提供现有的注册信息，包括注册号 | | | | |
| 纳入排除标准 | 6 详细说明作为纳入排除标准的研究特征（如PICOS和随访时间）和报告特征（如发表年份、语言和发表状态），并给出理由。  6a.1^●^描述目标疾病的西医诊断标准。  Line 138 - 144 in page 6.  6a.2^●^如果适用，描述目标疾病在传统医学中的诊断标准，例如中医。  6b^●^描述拟纳入的具体针刺类型，如手法针刺、电针或火针等。  6c^●^如果适用，描述拟关注结局指标在传统医学中（例如症状缓解得分）或西医中（例如疼痛强度量表和VAS量表）的评估或分类标准或工具。  Line 158 - 165 in page 6 - 7. | | | | |
| 检索信息来源 | 7*描述检索的所有信息来源（例如，数据库、检索时间范围、是否联系研究作者确定是否存在更多相关研究），并报告最后检索的日期。如果适用，报告检索的针刺或传统医学相关的数据库或补充检索方法。  Line 146 - 157 in page 6 – 7. | | | | |
| 文献检索 | 8*提供至少一个常用数据库（例如MEDLINE）完整检索策略，包括所有限定条件，以保证检索方法的可重复性。当系统评价同时检索了常用综合数据库和传统医学数据库时，则至少提供一个常用综合数据库和一个传统医学数据库完整检索策略。  The full PubMed search strategy is available in Supplementary Table 1. | | | | |
| 研究筛选过程 | 9 说明纳入研究的筛选过程（包括初筛、合格性判断及纳入系统评价等步骤，还可包括纳入Meta分析的过程）。  Line 145 - 170 in page 6 - 7. | | | | |
| 数据提取过程 | 10 描述资料提取方法（如设计提取表格、独立提取、重复提取）以及任何向纳入研究作者获取或确认资料和数据的过程。  Line 171 - 183 in page 7. | | | | |
| 数据提取条目 | 11*列出并定义要搜集的所有数据类型（如PICOS和资金来源）；如适用，描述参考用于制订数据提取表的工具（如STRICTA和TIDieR），对于干预措施信息的提取，针刺干预组和对照（如假针刺）的详细程度应该一致。  Line 171 - 183 in page 7. | | | | |
| 单个研究偏倚 | 12 描述用于评价单个研究偏倚风险的方法（包括该方法是用于研究层面或结局层面），以及在资料合并中该信息如何被利用。  Line 184 - 190 in page 7 – 8. | | | | |
| 概括效应指标 | 13 说明主要的综合结局指标，如相对危险度（risk ratio）、均值差（mean difference）。  Line 192 - 202 in page 8. | | | | |
| 结果综合 | 14 描述结果综合的方法，如果进行了 Meta 分析，则说明异质性检验方法（如 I2）。  Line 192 - 209 in page 8. | | | | |
| 研究偏倚 | 15 详细评估可能影响数据合并结果的偏倚（如发表偏倚和研究中的选择性报告偏倚）。  Line 203 - 209 in page 8. | | | | |
| 其他分析 | 16 描述研究中的其他分析方法，如敏感性分析或亚组分析，Meta回归分析等，并说明哪些分析是预先计划的。  Line 210-216 in page 8 - 9. | | | | |
| ***结果*** | | |  |  | |
| 研究筛选结果 | | | 17 报告文献筛选的相应数量，包括每个一步骤排除的文献数量和原因，最终纳入文献的数量，最好提供清晰的流程图。  Line 219 - 226 in page 9. | | |
| 研究特征 | | | 18*对于每项研究，报告提取的研究特征（例如，研究样本量、PICOS、随访时间）并提供所纳入研究的引文。参考TIDieR模板和STRICTA总结针刺干预特征，将每项研究的针刺干预细节总结在表格中。  18a^●^报告所纳入研究对实施针刺后出现的典型针刺感应（即“得气”）的描述情况。  Line 227 - 251 in page 9 - 10. | | |
| 单个研究偏倚 | | | 19 报告每个研究中可能存在的偏倚风险评价结果和相关信息，如果条件允许，还需要说明结局层面的评价结果。  Line 252 - 258 in page 10. | | |
| 单个研究结果 | | | 20 针对所有结局指标（有效性或安全性），说明每个研究中各个干预组结果的简单合并（a)，以及综合效应值及其可信区间（b)，最好以森林图的形式报告。  Figure 3 – 5. | | |
| 结果综合 | | | 21 报告每个Meta分析的结果，包括可信区间和异质性检验结果。  Line 259 - 296 in page 10 - 12. | | |
| 研究间的偏倚 | | | 22 报告对研究间可能存在的偏倚评价结果，如发表偏倚。  Line 297- 311 in page 12. | | |
| 其他分析 | | | 23 如果做了其他分析，则报告其他分析的结果，如敏感性分析，亚组分析和Meta回归分析等。 | | |
| ***讨论*** | | | Line 312 - 323 in page 12 - 13. |  | |
| 证据总结 | | | 24 总结研究的主要发现，包括每个主要结局的证据强度；分析结果与主要利益集团的关系，如医疗保健提供者和政策制订者。  Line 325 -434 in page 13 - 17. | | |
| 局限性 | | | 25 探讨研究层面和结局层面的局限性（如偏倚风险），以及系统评价的局限性（如检索策略不全面，存在报告偏倚等）  Line 436 -450 in page 17. | | |
| 结论 | | | 26 给出对结果的概要性解析，并提出对未来研究的启示意义。  Line 451-455 in page 17. | | |
| ***资金支持*** | | |  |  | |
| 资金支持 | | | 27 描述系统评价和Meta分析制作过程中的资金和其他支持（如提供资料）来源以及资助者在完成系统评价中的作用。  Line 464 -468 in page 18. | | |
